# An uncommon cause of ascites: uroperitoneum from iatrogenic bladder fistula detected by CT urography

**DOI:** 10.1259/bjrcr.20150391

**Published:** 2016-05-08

**Authors:** Carmelinda Manna, Mario Silva, Silvia Eleonora Gazzani, Rocco Cobelli

**Affiliations:** Sezione di Radiologia, Dipartimento di Scienze Chirurgiche, Ospedale Universitario di Parma, Parma, Italia

## Abstract

We report the case of a female who underwent laparoscopic hysterectomy and was referred to the emergency department with massive ascites 10 days later. Anamnestic background and clinical presentation suggested the occurrence of a urinary lesion, which was investigated by CT urography. CT urography with ultra-late excretory phase showed the leakage of iodinated contrast agent from the bladder dome into the peritoneal cavity, as expected in uroperitoneum from iatrogenic bladder laceration. CT cystography is the reference standard for the assessment of bladder leakage; however, this technique is quite invasive, time consuming and does not provide a panoramic overview of the entire excretory system. Conversely, CT urography provides a complete overview of the entire excretory system by means of an optimized protocol with optional ultra-late acquisition to gain adequate bladder distension and depict minor urinary leakage.

## Summary

We report the case of a female who underwent laparoscopic hysterectomy and was referred to the emergency department with massive ascites 10 days later. Anamnestic background and clinical presentation suggested the occurrence of a urinary lesion, which was investigated by CT urography. CT urography with ultra-late excretory phase showed the leakage of iodinated contrast agent from the bladder dome into the peritoneal cavity, as expected in uroperitoneum from iatrogenic bladder laceration. CT cystography is the reference standard for the assessment of bladder leakage; however, this technique is quite invasive, time consuming and does not provide a panoramic overview of the entire excretory system. Conversely, CT urography provides a complete overview of the entire excretory system by means of an optimized protocol with optional ultra-late acquisition to gain adequate bladder distension and detect minor urinary leakage.

## Clinical presentation

A 41-year-old female underwent radical laparoscopic hysterectomy for uterine cervical cancer. After the surgery, she was treated with administration of analgesic and antibiotic therapy for mild strangury and post-operative hypogastric pain. 1 week after discharge, the patient was referred to the emergency department with progressive abdominal swelling and hypogastric pain.

Vital signs at triage were heart rate 90 beats min^–1^, blood pressure 100/60 mmHg, respiration 18 breaths min^–1^ and body temperature 36°C. Physical examination revealed abundant distension of the abdomen, with tenderness on palpation. Laboratory tests were as follows: white blood cell count 8.42 × 10^3^ μl^−1^ (laboratory range 3.5–10.5 × 10^3^ μl^−1^), haemoglobin 12.4 g dl^−1^ (laboratory range 13.5–17.5 g dl^−1^), urea 56 mg dl^−1^ (laboratory range 20–40 mg dl^−1^) and serum creatinine 1.3 mg dl^−1^ (laboratory range 0.8–1.3 mg dl^−1^).

## Imaging findings/investigations

**Figure 1.**
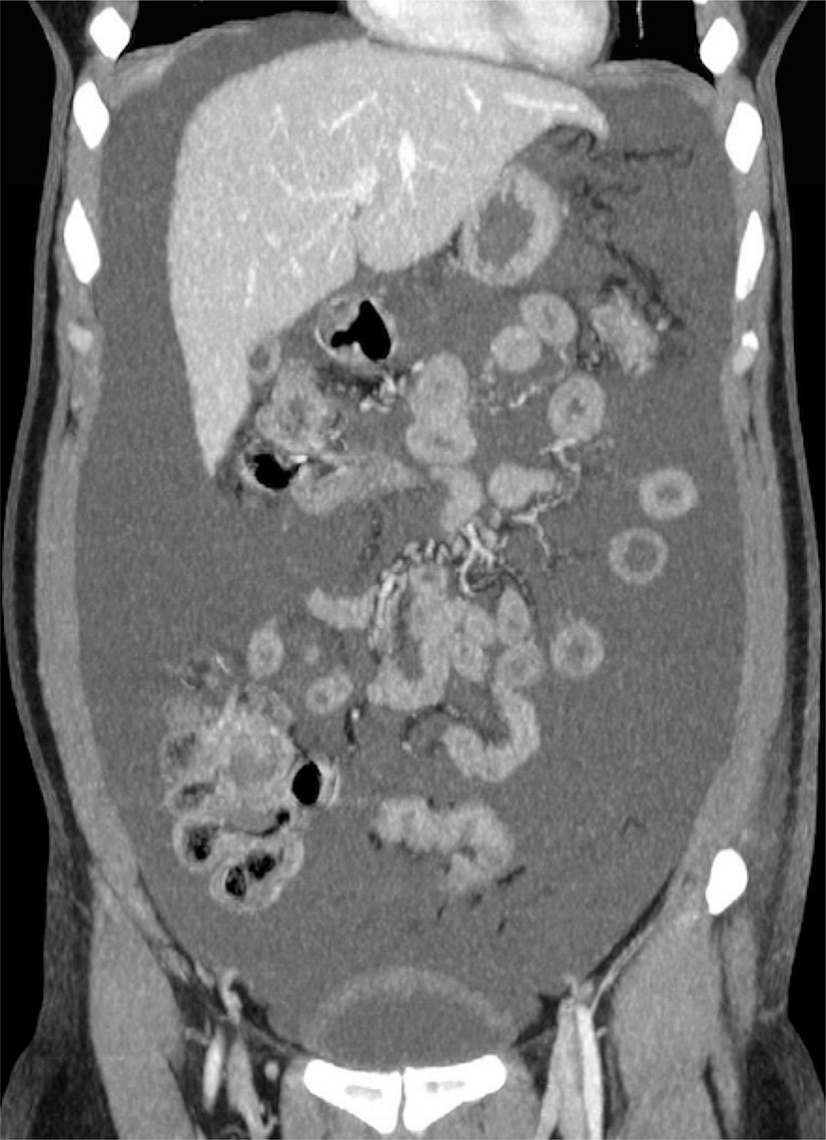
Coronal CT reformatted image showing widespread fluid within the abdomen.

**Figure 2.**
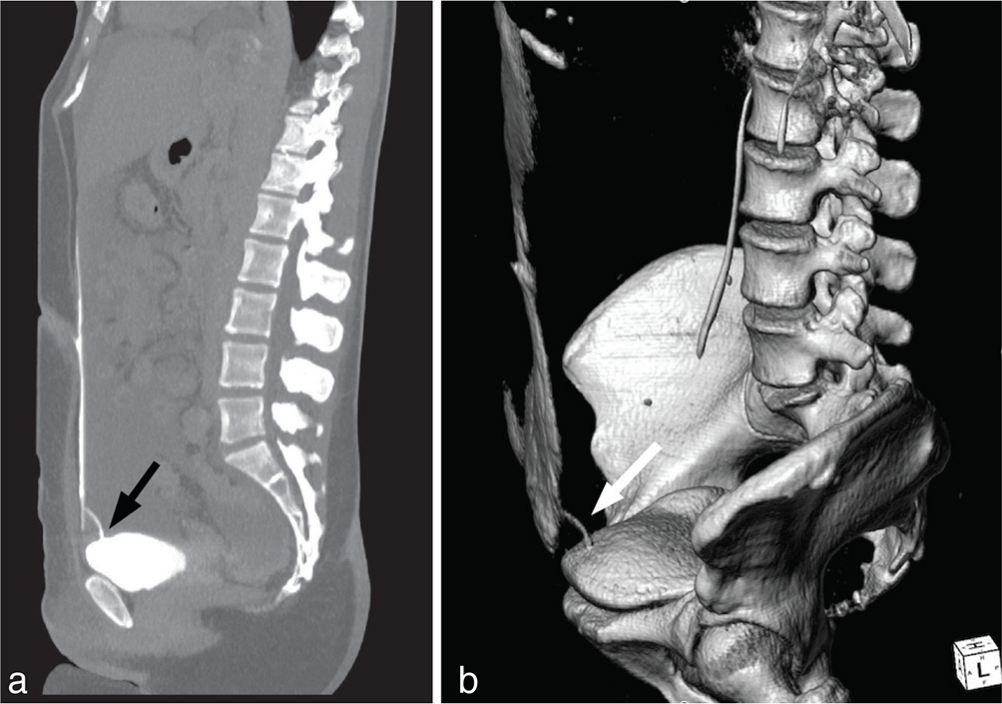
Sagittal CT maximum intensity projection image (a) and volume rendering technique reconstruction (b) both from the ultra-late scan (40 min after the injection of iodinated contrast agent) showing the tearing of contrast agent from the dome of the urinary bladder (arrows).

Ultrasonography of the abdomen revealed free fluid in the peritoneal cavity, estimated at about 3 l. Therefore, CT scan was performed, before and after injection of iodinated contrast agent (Iomeron 300, Bracco, Milan, Italy; injected volume 120 ml, injection rate 3 ml s^−1^), notably with urographic phase 10 min after the injection for specific assessment of post-surgical complication of the urinary tract. Massive ascites were confirmed; however, no further pathological finding was detected (Figure 1). Nevertheless, the temporal proximity between gynaecologic surgery and ascites onset was strongly associated with a suspicion of urinary system lesion. Therefore, the CT acquisition was completed with an ultra-late excretory scan in prone decubitus, 40 min after the injection of contrast agent. The ultra-late excretory scan showed leakage of contrast agent from the bladder dome into the peritoneal cavity, suggesting uroperitoneum due to urinary bladder laceration (Figure 2).

Laparoscopic examination revealed abundant citrinic fluid (3.5 l) and confirmed a pin-hole perforation in the dome of the bladder, which was treated by raffia. Cytological evaluation did not reveal neoplastic cells in the ascitic fluid. The patient’s condition improved after surgical reparation, and she was discharged on the 10th post-operative day.

## Discussion

Uroperitoneum is an uncommon cause of ascites, which should be suspected in patients with a history of surgery or radiation therapy for urinary bladder cancer or gynaecologic surgery.^1^ The urinary bladder is the most commonly injured organ during gynaecologic surgery, with a prevalence of 0.14–3.17% after radical hysterectomy.^2^ Urinary bladder perforation may be either extraperitoneal or intraperitoneal, with the latter being the most frequent presentation of iatrogenic injuries.^3^ In iatrogenic intraperitoneal perforation, direct CT signs (*e.g.* oedema surrounding the urinary bladder) are usually masked by post-surgical alteration. In this case, extravasation of contrast medium into the peritoneum (*e.g.* around bowel loops, between the mesenteric folds and in paracolic gutters) was the only reliable sign of perforation.

Prompt and accurate diagnosis of urinary bladder perforation is mandatory for appropriate management because delay in treatment increases morbidity and mortality. The diagnosis of iatrogenic bladder perforation is generally established intraoperatively and surgical repair is performed, with post-operative Foley catheter placement for 1–2 weeks. In some cases, urinary bladder perforation is not suspected in the subacute post-operative period because most patients initially show unspecific symptoms (*e.g.* suprapubic or abdominal pain, inability to void and progressive abdominal swelling).

Conventional cystography has been for a long time considered the reference standard for assessment of bladder injury. However, conventional cystography has been replaced by CT retrograde cystography, particularly for patients with traumatic rupture.^4^ Retrograde filling of the urinary bladder with diluted contrast agent prior to abdominopelvic CT scan is more sensitive than conventional cystography in describing the actual extent of urinary bladder injury.

Contrast-enhanced CT scan with excretory phase will not necessarily lead to identifying a small bladder rupture because a blood clot or the omentum may temporarily seal a small leakage, or bladder distension may not be adequate.^5^ It has been reported that filling the urinary bladder to ≥ 250–300 ml is necessary to confidently assess parietal tearing.^6^

In our case, the scan 10 min after the injection of contrast agent did not show any leakage. Conversely, a later scan with further urinary bladder distension and in the prone position favoured contrast tearing from the pin hole within the urinary bladder wall, and allowed detection of the leakage from the dome. Furthermore, CT urography provided a complete overview of the entire excretory system for comprehensive evaluation of ureteral anatomy.^7^ This is important because hysterectomies are also associated with up to 6% incidence of ureteral injury.^8^ CT urography is also relatively faster and less invasive than CT cystography.

In conclusion, instead of CT cystography, we suggest completing CT urography with an ultra-late scan in the prone position in case of suspicion of iatrogenic bladder lesions.

## Learning points

1. Uroperitoneum is an uncommon cause of ascites that should be particularly suspected after gynaecologic surgery.
2. Accuracy of CT urography can be increased by an optimized protocol with adequate urinary bladder distension and optional ultra-late scan in the prone position.

## Consent

Written informed consent was obtained from the patient for publication of this case report, including accompanying images.
